# Amyotrophic Lateral Sclerosis (ALS) and Adenosine Receptors

**DOI:** 10.3389/fphar.2018.00267

**Published:** 2018-04-16

**Authors:** Ana M. Sebastião, Nádia Rei, Joaquim A. Ribeiro

**Affiliations:** ^1^Instituto de Farmacologia e Neurociências, Faculdade de Medicina, Universidade de Lisboa, Lisbon, Portugal; ^2^Instituto de Medicina Molecular, Universidade de Lisboa, Lisbon, Portugal

**Keywords:** adenosine receptors, amyotrophic lateral sclerosis (ALS), spinal cord motor neurons, neuromuscular junction, glial cells

## Abstract

In the present review we discuss the potential involvement of adenosinergic signaling, in particular the role of adenosine receptors, in amyotrophic lateral sclerosis (ALS). Though the literature on this topic is not abundant, the information so far available on adenosine receptors in animal models of ALS highlights the interest to continue to explore the role of these receptors in this neurodegenerative disease. Indeed, all motor neurons affected in ALS are responsive to adenosine receptor ligands but interestingly, there are alterations in pre-symptomatic or early symptomatic stages that mirror those in advanced disease stages. Information starts to emerge pointing toward a beneficial role of A_2A_ receptors (A_2A_R), most probably at early disease states, and a detrimental role of caffeine, in clear contrast with what occurs in other neurodegenerative diseases. However, some evidence also exists on a beneficial action of A_2A_R antagonists. It may happen that there are time windows where A_2A_R prove beneficial and others where their blockade is required. Furthermore, the same changes may not occur simultaneously at the different synapses. In line with this, it is not fully understood if ALS is a dying back disease or if it propagates in a centrifugal way. It thus seems crucial to understand how motor neuron dysfunction occurs, how adenosine receptors are involved in those dysfunctions and whether the early changes in purinergic signaling are compensatory or triggers for the disease. Getting this information is crucial before starting the design of purinergic based strategies to halt or delay disease progression.

## Introduction

In the present review, we will address the potential role of adenosine on amyotrophic lateral sclerosis (ALS), also known as Lou Gehrig’s disease. This is one of the most devastating neurodegenerative disorders and is the most common form of Motor Neuron Diseases group. During ALS progression, both the upper motor neurons (motor neurons in the cortex) and the lower motor neurons (motor neurons in the spinal cord) degenerate or die. As a consequence of deficient input from the motor neuron, there is a progressive and terminal atrophy of skeletal muscles. All muscles under voluntary control are affected, and individuals lose their strength and the ability to move their arms, legs and body. When diaphragm and the muscles in the chest wall fail, people lose the ability to breathe without ventilation support (Nichols et al., 2013).

The incidence of this disease is 2–3/100,000 and the prevalence 4–7/100,000 (Nalini et al., 2008). The onset of symptoms is predominantly between 55 and 65 years old and the median survival of ALS patients, after the diagnosis, is 3–5 years; however, about 10% of the patients survive for 10 or more years (Wijesekera and Leigh, 2009).

The majority of cases of ALS are sporadic (sALS) (90–95%), but some cases have a positive familial history (fALS) (5–10%) (Kiernan et al., 2011). Both forms, sALS and fALS, present similar pathological and clinical features. In the case of fALS, the inheritance is autossomal dominant, but the penetrance is variable. About 20% of fALS are caused by a missense mutation in *SOD1* gene, encoding for the Cu/Zn superoxide dismutase 1 enzyme. This finding led to the first and most used rodent model for ALS, the SOD1^G93A^ mouse, which in the symptomatic phase recapitulate most features of the disease, including neuromuscular dysfunction (Naumenko et al., 2011). There is still a debate whether motor neuron degeneration initially results from failure of enzymatic machinery at the level of the cell body and proximal parts of the axon that then propagates in a centrifugal way due to impaired axonal transport (Braak et al., 2013), or results from an early dysfunction at the level of the nerve terminals, with consequent synaptic dysfunction and then progressing in a ‘dying back’ process (Frey et al., 2000; Dadon-Nachum et al., 2011), or both (Baker, 2014). As pointed out (Dadon-Nachum et al., 2011), if cell nerve terminal degeneration precedes with axonal and cell body degeneration, early intervention focusing on motor neurons terminals could potentially delay or prevent the progressive loss of motor neurons. A functional study focusing on the activity of single end-plates demonstrated that neuromuscular transmission impairment starts long before symptomatic onset (Rocha et al., 2013). Importantly, in pre-symptomatic SOD^G92A^ mice there are functional hallmarks of dysregulated intraterminal calcium levels (Rocha et al., 2013) in consonance with the hypothesis that nerve terminal machinery designed to buffer calcium might be impaired at very early disease stages. The pathophysiological alterations at the neuromuscular junction of SOD^G92A^ mice are accompanied with alterations in the activity of adenosine receptors at the motor nerve terminals in these mice (Nascimento et al., 2014, 2015).

Mutations in the gene coding for a nuclear protein, TAR DNA-binding protein-43 are also frequent in fALS (Leblond et al., 2014). This protein has several functions in regulation of gene expression, which thus indicates a clear dysfunction at the level of the neuronal soma in some forms of ALS. ROS induce the mislocalization of TAR-DNA binding protein 43 (TDP-43) from the nucleus into the cytoplasm (Ayala et al., 2011), where it forms aggregates, an early hallmark of ALS (Neumann et al., 2006; Chou et al., 2018). Cytoplasmic mislocalization of TDP-43 is accompanied by further ROS production and overactivation AMP kinase (AMPK), an action suppressed by A_2A_R activation (Liu et al., 2015a). Importantly, A_2A_R activation in a TDP-43Tg mouse model of ALS, improved motor function (Liu et al., 2015a). Interestingly also, activation of AMPK changed the location of a mRNA stabilizer in the motor neurons of ALS patients, in mouse motor neurons, and in a motor neuron cell line, and this mislocalization was also suppressed by activation of A_2A_Rs (Liu et al., 2015b). This again reinforces the idea of a putative neuroprotective role of A_2A_Rs in ALS since impaired RNA homeostasis is a major pathway for ALS pathogenesis (Liu et al., 2017).

Mutations in *C9ORF72* are also frequently found in fALS and accounts for 10% of the sporadic cases (DeJesus-Hernandez et al., 2011; Smith et al., 2013). Motor neurons with *C9ORF72* mutations have increased expression and activity of NMDA receptors and calcium permeable AMPA receptors (Selvaraj et al., 2018; Shi et al., 2018), known to be targets for adenosine receptor-mediated modulation (Dias et al., 2012, 2013). No information so far exists on the possibility that adenosine receptor ligands may influence motor neuronal survival in this model of ALS.

Being adenosine a ubiquitous neuromodulator, affecting synaptic transmission at pre- post and non-synaptic levels, having adenosine receptors not only neuroprotective but also excitotoxic and neuroinflammatory actions, it is somehow unexpected that only relatively few studies concentrated their attention on the influence of adenosine on ALS progression. Evidence so far available highlights early dysfunctions of neuromodulation by adenosine and the ability to influence those dysfunctions through manipulation of adenosine receptors. In this review we will critically analyze data so far available.

## Pathophysiology of ALS

ALS is a complex, multifactorial and multi-systemic disease, the pathophysiological mechanisms of motor neurons degeneration remaining yet incompletely known. Some of these mechanisms include RNA dysfunction, protein misfolding and aggregation, marked neuromuscular junction abnormalities, immune system deficiency, mitochondrial dysfunction, neuroinflammation, cytoskeletal derangements, desregulation of growth factors, oxidative stress, axonal disruption and apoptosis, excitotoxity (induced by glutamate), activation of nucleases and proteases and abnormal calcium metabolism (Pasinelli and Brown, 2006; Costa et al., 2010; Chou et al., 2018). It is becoming clear that ALS is a disease that involves different cell types and the communication between them, the damage that occurs in each cell population contributing to ALS pathogenicity and phenotype, thus to the progression of the disease (Taylor et al., 2016). Indeed, non-motor neuron cells, like interneurons, astrocytes, microglia, Schwann cells, skeletal muscle cells and oligodendrocytes play a crucial role in motor neuron survival, and their dysfunction impact upon motor neuron degeneration. In practical terms, a major problem that is shared with many neurodegenerative diseases is that when patients are diagnosed, the neuronal degeneration, in the case of ALS motor neuron degeneration, has already started and progressed.

Functional and structural changes at the level of the endplate are major events in ALS. It has now been realized that the functional changes may precede not only the structural ones but also the onset of symptoms. Studies conducted in our lab (Rocha et al., 2013) to investigate transmission at the diaphragm neuromuscular junction in SOD1^G93A^ mice revealed that the during the pre-symptomatic phase there is an enhanced release of ACh from nerve terminals, as determined by the mean quantal content of end-plate potentials. Also in these neuromuscular junctions the frequency of giant miniature endplate potentials (GMEPPs) was markedly increased, suggesting that the ability of the nerve terminal machinery to control cytoplasmatic [Ca^2+^] is dysregulated. In early symptomatic (defined as moderate changes performance in the RotaRod test) SOD1^G93A^ mice two groups of neuromuscular junctions were identified, both of them co-existing in the same innervated diaphragm (Rocha et al., 2013): one group, designated as group A (SOD1a group), presented significant reduction of the mean amplitude of EPP, a significant reduction of the mean amplitude of MEPPS, and a slight but not significant reduction of the quantal content of EPPs. All these changes indicate a predominant post-synaptic impairment. The frequency of GMEPPs was significantly reduced in SOD1a neuromuscular junctions, even as compared with wild type siblings, indicating that presynaptic calcium dysregulations persisted, though with opposite signal in pre-symptomatic and in symptomatic mice. The other group of neuromuscular junctions, designated as group B (SOD1b) (Rocha et al., 2013) displayed electrophysiological characteristics somehow in between those of age-matched wild type and pre-symptomatic mice, being not significantly different from any of these groups. Summarizing, data suggest (Rocha et al., 2013) an exacerbated, but already dysregulated, presynaptic activity at the mice neuromuscular junctions that do not yet display motor symptoms; once symptoms start to appear some neuromuscular junctions are already hypofunctional, some others being probably in a transition between hyper and hypofunction.

Presynaptic facilitation of neuromuscular transmission preceding motor symptoms was further confirmed in using a late onset slow progressing disease model, the SOD1^G37R^ mice (Arbour et al., 2015). A recent study (Arbour et al., 2017) taking advantage of this late onset slow progressing disease model, systematically analyzed the changes in synaptic properties over the course of the disease progression and as a function of the motor unit type. Indeed, different motor units have different susceptibility to the disease, and it became clear the time course and the sequence of events associated with neuromuscular transmission dysfunction depend on the motor unit type (Arbour et al., 2017). The slow motor neurons are those that degenerate later and interestingly, they display an early and transient increase in the quantal content of endplate potentials that disappear at disease onset. In contrast, fast fatigue motor neurons, those that degenerate first, have reduced quantal content even before disease onset. Somehow in the between, fatigue resistant motor units only evidence neuromuscular transmission dysfunction after disease onset (Arbour et al., 2017). Interestingly only slow-type motor neurons display intrinsic hyperexcitability in pre-symptomatic mice (Leroy et al., 2014), pointing toward the possibility that early intrinsic hyperexcitability does not contribute to motor neuron degeneration, but rather represents an early compensatory process. It is worthwhile to note that as previously seen while using the SOD1^G93A^ mice (Rocha et al., 2013), in SOD1^G37R^ mice the neuromuscular transmission alterations precede the onset of motor symptoms, as well as precede motor neuron loss, axonal degeneration, and NMJ structural changes (Arbour et al., 2017).

It is nowadays clear that motor neuron degeneration in ALS also involves glial cells, namely astrocytes, oligodendrocytes, microglia, Schwann Cells and, in the case of the neuromuscular junction, perisynaptic Schwann cells (PSCs). PSCs have neuromodulatory properties close to those of astrocytes in the central nervous system, closely interacting with nerve terminals. Thus, PSCs respond to ATP and ACh released during nerve activity, which act through P2Y and muscarinic ACh receptors (mAChRs) coupled to transient increases in intracellular calcium concentration, a process that has to be kept under proper control to keep PSCs competent for their functions to modulate and to repair neuromuscular transmission (Son et al., 1996; Georgiou et al., 1999; Feng and Ko, 2008a,b). Interestingly, PSC of SOD1 mice displayed exacerbated mAChR responses and exacerbated calcium signaling, which precede functional and morphological changes at the neuromuscular junction, suggesting that the impairments of PSC functions may contribute to NMJ dysfunction and ALS pathogenesis (Arbour et al., 2015).

At the level of spinal cord and focusing in neuroinflammatory and cellular intercommunication hallmarks, it has recently been also shown a clear difference between pre-symptomatic and symptomatic stages (Cunha et al., 2017). Thus, before onset of motor symptoms, alterations in both astrocytes and microglia have been detected in the spinal cord of SOD1^G93A^ mice, which comprise decreased expression of astrocytic, microglia, inflammatory and cell communication markers together with upregulation of a glutamate transporter-1 marker. In contrast, in the symptomatic stage, increased markers of inflammation became evident (Cunha et al., 2017). Microglia activation, suggestive of a switch from M1 to M2-like microglia subpopulations, have been detected in control cells exposed to exosomes derived from motor neuron-like cells transfected with mutant SOD1^G93A^ (Cunha et al., 2017), thus suggesting that disease progression is promoted by cell to cell communication events. There is also a growing body of evidence showing that astrocytes expressing ALS-associated proteins impair motor neuron survival and potentiate ALS progression (Di Giorgio et al., 2007; Nagai et al., 2007; Marchetto et al., 2008; Qian et al., 2017).

Recent attention has been paid to two other ALS-related dysfunctions, TDP-43 aggregation and *C9ORF72* repeated expansion. Mutations in the gene that codes for TDP-43, *TARDBP*, may favor TDP-43 aggregation but phosphorylated TDP-43 containing aggregates also occur in sALS (Neumann et al., 2006; Chou et al., 2018). Indeed, TDP-43 aggregates are found in 97% of ALS cases of diverse etiology and constitute a major component of protein inclusions in this disease (Arai et al., 2006; Maekawa et al., 2009). TDP-43 is a nuclear protein that interacts with RNA molecules and is involved in a wide variety of relevant cellular pathways related to RNA and protein homeostasis. TDP-43 C-terminal missense mutations have been identified in ALS patients, which promote mislocalization of TDP-43 from the nucleus to the cytoplasm and lead to neurotoxicity (Gitcho et al., 2008; Nonaka et al., 2009; Guo et al., 2011). Oxidative stress and ROS formation also favor mislocalization of TDP-43, from the nucleus to the cytoplasm (Ayala et al., 2011), being thus not surprising that SOD mutations also lead to TDP-43 pathology in mice and humans (Shan et al., 2009; Pokrishevsky et al., 2012). ROS upregulates AMP kinase (AMPK) and abnormal AMPK activity has been shown to induce TDP-43 mislocalization in a motor neuron cell line and in the spinal cord of ALS patients (Liu et al., 2015a). Interestingly, mislocalized TDP-43 in the cytoplasm triggers a positive feedback loop, leading to further ROS production and AMPK activation (Liu et al., 2015a).

The present understanding of the mechanisms that underlie motor neuron degeneration associated to expansion of a G4C2 intronic hexanucleotide of the *C9ORF72* gene, is scarce. This mutation is a common cause of fALS but also accounts for near 10% of sALS cases (DeJesus-Hernandez et al., 2011; Smith et al., 2013). A recent study has shown that the *C9ORF72* mutation is associated with an increase in GluA1 AMPA receptor subunit expression, functional expression of Ca^2+^-permeable AMPA receptors and motor neuron vulnerability to excitotoxicity (Selvaraj et al., 2018). Interestingly, early intrinsic hyperexcitability has been detected in mixed cultures of neurons and glia derived from *C9ORF72* repeat expansion patient iPSCs (Wainger et al., 2014), but not in neuronal cultures with negligible number of glial cells (Selvaraj et al., 2018). Also, conditional medium from astrocytes expressing mutant SOD1 can induce motor neuron hyperexcitability (Fritz et al., 2013). Altogether these studies reinforce the idea that diseased astrocytes trigger excitotoxicity.

Motor neurons, astrocytes, microglia and perisynaptic Schwann cells are known to release and respond to purines, namely ATP and adenosine. Adenosine, by activating membrane receptors (A_1_, A_2A_, A_2B_, A_3_), is an endogenous and ubiquitous modulator of synaptic signaling. Released ATP is a source for extracellular adenosine, but acts on its own purinergic P2 (P2Y and P2X) receptors. So the challenging question is: How purinergic receptors affect the motor neurons and how this impacts in ALS? In this review we will only refer to adenosine receptors since the putative involvement of ATP receptors in the pathophysiology of ALS has been matter of a recent review (Volonté et al., 2016).

## Adenosine and Adenosine Receptors

Adenosine is a ubiquitous endogenous neuroprotective agent, which has a central role as a neuromodulator of synaptic transmission at the central and peripheral nervous systems, protecting organs and tissues at both physiological and pathophysiological conditions. Adenosine exerts its biological effect via a class of purinergic G protein coupled receptors, which includes A_1_R, A_2A_R, A_2B_R and A_3_R, all belonging to family of receptors named by Burnstock (1976) as P_1_ purinoceptors (adenosine-sensitive) as opposed to the ATP-sensitive P2 purinoceptors. The A_1_R couple to G_i/o_ proteins, inhibiting the production of cyclic AMP. The A_2A_ and A_2_B receptors couple to G_s_, stimulating the production of cyclic AMP, whereas the A_3_R may couple to G_i/o_ or G_q_ proteins. Adenosine acts like an extracellular signaling molecule, modulating the action of several neurotransmitters (Ribeiro et al., 2003).

Adenosine A_1_R are widely distributed in the brain having a widely recognized inhibitory action in synaptic transmission associated to a neuroprotective role, while A_2A_R have more restrict localizations, being predominant at the basal ganglia. However, both receptors are present in the cortex, the A_1_R being the predominant in this brain area (Dunwiddie and Masino, 2001; Sebastião and Ribeiro, 2009a). A_2A_R may exacerbate excitotoxicity in several brain areas (Ribeiro et al., 2016b) as well as in motor neurons (Mojsilovic-Petrovic et al., 2006). However, A_2A_R are also able to gate the action of neuroprotective molecules as the neurotrophic factors, a mechanism known to occur in the brain (Diógenes et al., 2004) as well as in motor neurons (Wiese et al., 2007). Trophic actions of A_2A_R in cortical neurons, some of them being independent of the interplay with neurotrophic factors, have been recently identified (Ribeiro et al., 2016a). Expression of A_2A_R in spinal motor neurons is intense, being higher than that of A_1_R (Kaelin-Lang et al., 1999; Ng et al., 2015).

A_1_R in the spinal cord inhibit excitatory inputs (Deuchars et al., 2001) whereas A_2A_R facilitate inhibitory inputs (Brooke et al., 2004) to sympathetic preganglonic neurons, suggesting that at least in what concerns autonomic nervous system control, the opposing actions of A_1_R and A_2A_R at the synaptic level may both contribute to a common outcome – a decrease in preganglionic neuron activity. The antinociceptic actions of adenosine A_1_R are widely known, which involve A_1_R at the dorsal horn of the spinal cord. A_3_R receptors also mediate antinociception through spinal and supraspinal mechanisms (Little et al., 2015) that also involve anti-inflammation (Janes et al., 2015).

Concerning motor pattern generation, it has been for a long time described that ATP and adenosine have opposite actions in motor pattern generation, the adenosine actions being inhibitory, lowering the excitability of motor circuits (Dale and Gilday, 1996). Recent studies have shown that adenosine, via A_1_R, has a general inhibitory action on ventral horn interneurons while potentially maintaining motor neuron excitability (Witts et al., 2015). Interestingly, glial cells are a predominant source of adenosine at to control motor neuron networks at the spinal cord (Witts et al., 2012; Carlsen and Perrier, 2014; Acton and Miles, 2015).

The motor nerve terminals at the neuromuscular junction have served as a model to the pioneer studies on the inhibitory action of adenosine and ATP on neurotransmitter release (Ginsborg and Hirst, 1972; Ribeiro and Walker, 1973, 1975), being now well known that endogenous adenosine inhibits neuromuscular transmission (Sebastião and Ribeiro, 1985), that ATP is a source of extracellular adenosine at the endplate (Ribeiro and Sebastião, 1987), and that adenosine can also be independently released from stimulated motor nerve endings (Cunha and Sebastião, 1993). Motor nerve terminals at the mammalian skeletal neuromuscular junction possess both inhibitory A_1_R (Sebastião et al., 1990) and excitatory A_2A_R (Correia-de-Sá et al., 1991), the A_2A_R gaining particular relevance at high frequencies of nerve stimulation (Correia-de-Sá et al., 1996). A_2B_ and A_3_ receptors were also more recently detected at the mammalian neuromuscular junction (Garcia et al., 2014).

### Adenosine Receptors in Health and Disease

At physiological conditions, the adenosine exerts its neuromodulation action by the activation of A_1_ and A_2A_R high affinity receptors (Sebastião and Ribeiro, 2009a), which mediate dual actions of adenosine both in health and disease (Fredholm, 2014). At pre-synaptic level, adenosine can inhibit or facilitate transmitter release through A_1_R and A_2A_R respectively; at post-synaptic level, it can modulate the actions of several neurotransmitters and it can hyperpolarize or depolarize neurons. All these receptors can be found in neurons and glia cells. While the A_1_R is frequently associated to neuroprotective actions, which extend to conditions as hyperexcitability, seizures and ischemia/hypoxia, protecting neurons in response to excitotoxic injury (Boison, 2016; Cunha, 2016; Ribeiro et al., 2016b), the A_2A_R is often associated to enhancement of excitotoxicity, since these receptors facilitate glutamate release and inhibit glutamate uptake (Popoli et al., 2003; Matos et al., 2012). However, through their ability to gate the actions of neurotrophic factors, A_2A_R may confer a protective role under specific pathologic conditions (Sebastião and Ribeiro, 2009b; Rodrigues et al., 2014). A_1_R, A_2A_R and A_2B_R can also influence oxygen deliver into the brain due to their influence in neurovascular coupling (Pelligrino et al., 2010).

Concerning inflammation, A_2A_R have a duality of actions, depending on the cell type and inflammatory conditions (Haskó et al., 2008). In immune cells the global trend for A_2A_R-mediated actions is to interrupt the proinflammatory cascade and hence limiting tissue inflammation (Sitkovsky et al., 2004; Haskó et al., 2008), contrasting with A_2_B receptors that may promote overproduction of proinflammatory cytokines (Haskó et al., 2008). In the central nervous system, however, A_2A_R mediate anti-inflammatory effects on T cells, and thus protection at early stages of inflammatory diseases; during later stages of disease, however, may contribute to sustained tissue damage within the inflamed central nervous system (Ingwersen et al., 2016). In microglia, A_2A_R activation leads to their activation, and in such way contributing to foster neuroinflamatory cascades, thus to neurodegeneration (Dai and Zhou, 2011; Rebola et al., 2011; Santiago et al., 2014). Similarly, A_2B_R and A_3_R may mediate either anti-inflammatory or pro-inflammatory actions (Borea et al., 2017).

### Adenosine Receptors in ALS

#### Adenosine Receptors at Spinal Cord: Implications for ALS

In accordance with the expected facilitatory influence of A_2A_R upon excitotoxicity, A_2A_R antagonists protect against excitotoxicity-induced motor neuron death (Mojsilovic-Petrovic et al., 2006). However, motor neuron survival after mechanical lesioning has also been shown to be increased by an A_2A_R activation, through a mechanism that involves interplay with neurotrophines (Wiese et al., 2007). Survival of motor neurons in culture is also facilitated by A_2A_R activation, this mechanism requiring the activity of the canonical A_2A_R signaling pathway, cyclic AMP/protein kinase activity, as well as the activity of neurotrophin receptors, again highlighting an interplay between A_2A_R and neurotrophins to promote motor neuron survival (Komaki et al., 2012). The apparent discrepancies between the neuroprotective actions of A_2A_R antagonists and A_2A_R agonists probably result from the diversity of actions that the A_2A_R have, namely modulation of neuronal activity, neuronal survival, excitotoxicity, neuroinflammation. While facilitating the actions of neurotrophic factors A_2A_R may promote survival and regeneration, but while enhancing excitotoxicity phenomena A_2A_R will favor neuronal death. In addition, if neuroinflammation interferes with in the regeneration/degeneration balance A_2A_R may again have a dual role, depending on the degree and characteristics of the inflammatory process, that it to say, if activation of the inflammatory cascade is beneficial or detrimental. Unraveling all these aspects by taking into consideration the nature of the insult and the time window for treatment is of uttermost importance to understand the role of adenosine receptors in neurodegeneration and in particular in ALS. Our poor understanding of the pathophysiology of the disease itself makes it more difficult to understand the role of adenosine receptors in this disease.

Adenosine levels are significantly elevated in the cerebrospinal fluid of ALS patients (Yoshida et al., 1999), a finding that raised the interest on the understanding the role of adenosine and adenosine receptors in ALS. The expression of A_2A_ receptors is also enhanced in lymphocytes of ALS patients (Vincenzi et al., 2013) as well as in post-mortem spinal cord of ALS patients (Ng et al., 2015). A_2A_R expression in the spinal cord of early symptomatic SOD1^G93A^ mice was also markedly increased (Ng et al., 2015), but in end-stage SOD1^G93A^ mice a marked decrease was reported (Potenza et al., 2013). Mice data thus may suggest that there is an early enhancement of A_2A_R expression in the spinal cord, followed by a decrease in latter disease states. A comparison of data obtained in the same laboratory conditions and same animal housing conditions is, however, needed to firmly conclude on biphasic changes in A_2A_R expression during disease progression. Post-mortem human data (Ng et al., 2015), by definition related to end-stages of the disease, seems to contradict the conclusion of a late-phase decrease in A_2A_R expression in the spinal cord. Whether the discrepancy arrives from methodological issues (post-mortem time of analysis, tissue collection procedures), species differences (mice vs. human) or from inappropriate disease model, is unknown.

Caffeine is a non-selective adenosine receptors antagonist (Pelligrino et al., 2010; Ribeiro and Sebastião, 2010; Fredholm et al., 2017) and is one of the most consumable psychoactive substances of the western diet. There is thus high interest in understanding the actions of caffeine in neurodegenerative diseases. Indeed, caffeine consumption in humans has been negatively associated with the incidence of some neurodegenerative diseases, as Alzheimer’s Disease and Parkinson’s Disease (de Mendonça and Cunha, 2010; Flaten et al., 2014; Laurent et al., 2014; Ascherio and Schwarzschild, 2016). Accordingly, chronic caffeine consumption has been shown to protect several hallmarks of neurodegeneration in animal models of disease, these actions being frequently mimicked by selective A_2A_R antagonists or A_2A_R deletion (Canas et al., 2009; Kachroo and Schwarzschild, 2012; Laurent et al., 2016; Ferreira et al., 2017). Concerning ALS, however, conflicting data had recently emerged in the literature, and there are several points that are far from being understood. In clear contrast to what could be expected from previous evidence in other neurodegenerative diseases, chronic administration of caffeine significantly shortens the survival of SOD1^G93A^ mice (Potenza et al., 2013). Also somehow unexpected, chronic administration of caffeine caused a marked decrease in A_2A_R levels in the spinal cord (Potenza et al., 2013) and in this aspect seems to mimic the disease itself since SOD1^G93A^ mice also displayed a strong reduction in A_2A_R in the spinal cord. Both conditions (caffeine treatment and SOD^G93A^ mutation) were not additive since there were no further reduction in spinal cord A_2A_R levels in SOD1^G93A^ mice treated with caffeine (Potenza et al., 2013). Caffeine can inhibit all adenosine receptors, and thus one cannot preclude that those actions of caffeine were due to antagonism of the usually neuroprotective A_1_R rather than antagonism of the A_2A_R. Indeed, A_1_ and A_2A_R are known to strongly interact with each other (Ferre et al., 2008), including in motor neurons (Pousinha et al., 2010, 2012), and the loss of inhibitory control of A_1_R upon A_2A_R may cause marked unbalance of the fine-tuning of neuronal function exerted by adenosine receptors. A selective A_2A_R antagonist, KW6002, administered daily, was recently reported to increase of motor neuron survival, delay the onset of disease and increase in the lifespan that SOD1^G93A^ mice (Ng et al., 2015), which is in accordance with the usual finding that A_2A_R may exacerbate excitotoxicity.

Concerning the influence of A_2A_R agonists in ALS progression, it has been shown that treatment of SOD1^G93A^ mice with CGS21680 at a very early symptomatic stage of the disease, delayed disease onset of motor symptoms and enhanced motor neuron survival (Yanpallewar et al., 2012). Interestingly, the effect of the A_2A_R agonist was similar to that resulting from genetic deletion of the truncated form of the TrkB receptor, TrkB.T1, which is overexpressed in the spinal cord of the SOD1^G93A^ mice. The TrkB.T1 act as negative regulator of full-length TrkB receptor signaling, impairing the ability of its ligand, the neurotrophin Brain Derived Neurotrophic factor (BDNF), to promote neuronal survival, neuronal maturation and neuronal plasticity. Most of the actions of BDNF at synapses are triggered by A_2A_R activation (Diógenes et al., 2004; Sebastião et al., 2011; Ribeiro et al., 2016b). It is thus likely that the protective influence of A_2A_R receptor agonist in very early stages of ALS progression in SOD^G93A^ mice results from the ability of A_2A_R to facilitate full length TrkB receptor activation, thus compensating the negative influence the TrkB.T1 receptor overexpression. That A_2A_R and A_2A_R antagonists may have strict time windows to exert its beneficial influences in neurodegeneration has been already proposed (Sebastião and Ribeiro, 2009b) but data from experiments specifically addressing this point have not yet appeared.

Also particularly relevant in the context of understanding the role of A_2A_R in ALS progression was the finding that A_2A_R activation, through PKA signaling, suppresses aberrant AMPK activity as well as suppresses mislocalization of TDP-43, in the TDP-43 mouse model of ALS (Liu et al., 2015a). Taking into consideration that mislocalization of TDP-43 from the nucleus to the cytoplasm can be induced not only by mutations in the gene that codes for TDP-43 but also by ROS production, there is some overlap in the disease triggering mechanisms in the SOD mouse models and in the TDP-43 model.

Summarizing, in two models of ALS (SOD1^G93A^ and TDP-43), two different A_2A_R agonists proved beneficial to improve motor neuron survival (Yanpallewar et al., 2012; Liu et al., 2015a). One study clearly showed detrimental effects of the adenosine receptor antagonist, caffeine in the survival of SOD1^G93A^ mice (Potenza et al., 2013). Thus, as whole and in the specific context of ALS, A_2A_R activation seems beneficial. Importantly, A_2A_R, through cAMP/PKA signaling and subsequent inhibition of AMPK may contribute to cut the positive feedback mechanism where AMPK activation by ROS leads to further mislocalization of TDP-43, which leads to further ROS production, thus to further AMPK activation. In this way, A_2A_R activation may hit a key pathophysiologic mechanism of ALS, thus reducing or even halting disease progression. Unfortunately, in the study by Liu et al. (2015a), the influence of A_2A_R activation in the time of appearance of first symptoms or in the life-span of ALS mice was not assessed. Results from such studies would indeed complement the evidence already obtained on enhancement of motor neuron survival by A_2A_R activation in two models of ALS (Yanpallewar et al., 2012; Liu et al., 2015a). Also, and taking into consideration that the agonist used by Liu et al. (2015a) already entered clinical trials for other neurodegenerative diseases, a clinical trial specifically designed for a fast devastating disease as ALS, would be of high relevance.

A_2A_R and D_2_ dopamine receptors (D_2_R) co-exist in motor neurons of the spinal cord of normal subjects and ALS patients (Chern et al., 2017). Activation of D_2_R reverse the A_2A_R-mediated prevention of ROS-induced AMPK activation and TDP-43 mislocalization in cells co-transfected with D_2_R and A_2A_R (Chern et al., 2017). A_2A_/D_2_ negative interactions are well known in the basal ganglia (Fuxe et al., 2007). The evidence that they also exist at the level of the spinal cord and in a context of ALS pathophysiology (Chern et al., 2017), point toward the need of care in the use of D_2_R agonists in ALS patients, as well as may suggest a beneficial role of D_2_R antagonists in this disease.

From the studies mentioned above, it appears that A_2A_R agonists may prove of therapeutic value in ALS. Yet, there are several discrepancies in the literature, as the report that a selective A_2A_R antagonist may delay disease progression. To solve some discrepancies, it will be important to compare in the same colony and under the same experimental conditions, and always against age-matched control littermates, time-dependent and disease state-dependent changes in the activity and expression of adenosine receptors. The comparisons among different mouse models are also of uttermost importance to allow starting to understand the different roles of A_2A_R as a function of the etiology of the disease itself. It will be also necessary to follow those changes in mice treated with either selective receptor ligands as well as with caffeine. Since ALS progression may not be similar at different synaptic levels, a better understanding of the time-dependent changes in A_2A_R signaling at upper and lower motor neuron levels would also allow a better understanding in the role of A_2A_R in ALS. Information of the role of A_1_R in this neurodegenerative disease is scarce, which is somehow surprising on the light of the known neuroprotection exerted by these receptors. Their ability to inhibit synaptic transmission, may, however, exert a negative influence in a degenerative disease that markedly compromises information flow in the motor circuit.

#### Adenosine Receptors at the Neuromuscular Junction: Implications for ALS

The neuromuscular junction early served as a model to understand synaptic transmission mechanisms (Katz and Miledi, 1965), as model to understand neuromodulation by adenosine (Ginsborg and Hirst, 1972), as a model to understand the relevance of endogenous adenosine to control synaptic transmission (Ribeiro and Sebastião, 1987), and as a model to start identifying A_2A_R at synapses outside the basal ganglia (Correia-de-Sá et al., 1991; Correia-de-Sá and Ribeiro, 1994). Reasons for this are the simplicity of the model, allowing to record from a single synapse receiving input from only one nerve terminal, with a clearly identified neurotransmitter - acetylcholine. In spite of this simplicity, there are several neuromodulators released by either the pre-synaptic, the post-synaptic, the muscle itself and the perisynaptic Schwann cells, which finely tune synaptic transmission and synaptic maintenance. As widely known, ATP and adenosine are among such neuromodulators. Neurotrophic factors are also present at the neuromuscular junction and A_2A_R are also indispensable promoters of the facilitatory action BDNF, upon neuromuscular transmission (Pousinha et al., 2006), as it was firstly observed hippocampal synapses (Diógenes et al., 2004).

Having studied the action of adenosine for more than 40 years, using the neuromuscular junction as a model, and considering that this simple synaptic model could be as a sort of key (Arbour et al., 2017) to investigate novel approaches for disease therapy, and also that this particular synapse is compromised in ALS, we hypothesized (Nascimento et al., 2014, 2015) that ALS progression could be accompanied by changes in the functioning of adenosine A_1_R and A_2A_R, the two high affinity adenosine receptors known to be expressed at motor nerve endings (Correia-de-Sá and Ribeiro, 1994). We used a functional approach, recording endplate potentials (EPPs) and miniature endplate potentials (MEPPs), and quantifying quantal content of EPPs, from Mg^2+^-paralyzed hemidiaphragm preparations of SOD1^G93A^ mice at pre-symptomatic (absence of motor symptoms assessed by the Rota-Rod) and early symptomatic (mild but significant dysfunction at the Rota-Rod) phases of the disease. We found that at the pre-symptomatic stage the A_2A_R-mediated presynaptic facilitatory action on neuromuscular transmission is exacerbated, whereas in the early symptomatic phase, this excitatory action disappears, indicating that indeed that A_2A_R function changes upon ALS progression. We then further investigated the role of A_1_R in ALS using the same approach and the same disease model (Nascimento et al., 2015) and found that the A_1_R/A_2A_R functional cross-talk is lost in the pre-symptomatic phase, so that the ability of A_1_R to brake the action of A_2A_R is lost, which might explain the exacerbation of the A_2A_R signaling at this disease state. In addition, there was an increase of A_1_R tonic activation in the symptomatic phase, suggesting that the changes adenosine modulation mediated by A_1_R may be contributing to disease progression and aggravating symptoms in late disease stage. Whether the lack of A_1_R-mediated inhibition of A_2A_R function, with the corresponding exacerbation of A_2A_R-mediated function, corresponds to an early compensatory process that may facilitate neuromuscular transmission and confer some neuroprotection through an enhancement of the action of neurotrophic factors, remains unknown. Also unknown is if these early excitatory changes, accompanied by dysruptions of the calcium buffering at the nerve terminal (Rocha et al., 2013) and at perisynaptic Schwann Cells (Arbour et al., 2015), are a fast-track to neurodegeneration.

**FIGURE 1:**
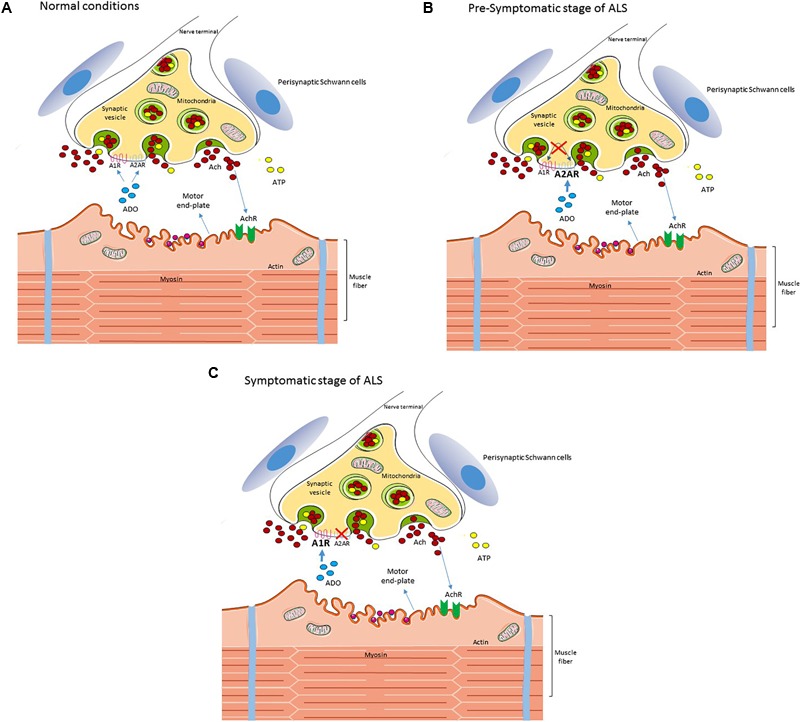
Schematic diagram of the reported changes in adenosine signaling at the neuromuscular junction in an ALS mice model. In wild type **(A)** neuromuscular junctions, adenosine activates both inhibitory A_1_R and excitatory A_2A_R, modulating acetylcholine release from motor nerve terminals. In ALS mice at the pre-symptomatic stage **(B)** there is a loss of A_1_R/A_2A_R functional cross-talk at the neuromuscular junction and the excitatory action of A_2A_R is exacerbated. At the symptomatic stage **(C)** there is an increase of A_1_R tonic activation, which may be contributing to neurotransmission failure on late disease stages, whereas the excitatory action of A_2A_R disappears. For details see Nascimento et al. (2014, 2015). Perisynaptic Schwann cells contribute to the pool of ATP at the synaptic cleft but ATP signaling in these cells seems unchanged in ALS, in contrast with mACh signaling (Arbour et al., 2015). Interestingly, the exacerbated A_2A_R signaling at the pre-symptomatic stage correlates with an enhanced release of ACh from nerve terminals and with signs of dysregulated calcium buffering at motor nerve endings (Rocha et al., 2013; Arbour et al., 2017).

Summarizing, data (Nascimento et al., 2014, 2015) suggest that in ALS there is an unbalanced adenosine receptors modulation at the neuromuscular junction (**Figure 1**) that may start before first signs of motor impairment. We anticipate early compensatory alterations followed by a disruption of neuromodulatory control that may then act as an aggravating factor to exacerbate excitotoxicity, fasting neuronal death. A clear identification of the nature of these adenosine receptors changes, at different diseases states, would allow to identify new therapeutic targets based on the cause of neurodegeneration and thus to halt it.

Interestingly, adenosine A_1_R at motor nerve endings, besides inhibiting the release probability, also synchronize release by removing quantal events with long latencies, an action that is sensitive to the redox potential, being abolished by oxidants (Tsentsevitsky et al., 2013). How this impacts in oxidative-stress related motor neuron diseases, as ALS, is yet unknown.

## Conclusion and Perspectives

A major finding in what concerns adenosinergic signaling in ALS is the existence of very early synaptic alterations, which precede motor symptoms and which are evident at the level of the neuromuscular junction as well as, most probably, at central synapses. Whether those changes are triggers for the disease or represent early compensatory modifications is yet unknown. The evidence of a positive influence of A_2A_R agonists and negative influence of caffeine, may suggest that an early enhancement of A_2A_R functioning may represent an endogenous attempt to fight the disease. The positive influence of a selective A_2A_R antagonist may, however, contradict this possibility. A better clarification of the nature of the purinergic signaling dysfunctions, their implications and their time-windows, is for sure relevant not only for a better understanding of the pathophysiology of the disease and the implications of caffeine intake, but also to guide the design of putative therapeutic strategies to halt disease progression. Specifically, it is important to know if the early exacerbation of A_2A_R signaling detected at the motor nerve endings also occurs at central synapses, in particular, at the spinal cord and motor cortex, and how selective blockade of these receptors at different disease states affects disease progression. Keeping in mind the intriguing data reported while testing the influence of caffeine in animal models, it is also of uttermost importance to understand the molecular basis of this action of caffeine, whether it has an impact in the signaling of other neuroprotective molecules, or in the signaling mediated by other adenosine receptors. This knowledge is not only important for the design of future drugs but also to guide research in patients since caffeine is present in many widely consumed beverages and is usually regarded as protective against other neurodegenerative diseases. Lastly, the putative influence of A_2A_R and A_3_R in the inflammatory cascade associated to ALS has to be better understood. Indeed, recent studies have demonstrated the presence of inflammation propagating substrates in the central nervous system of patients afflicted with ALS (Khalid et al., 2017). A_2A_R receptors mediate anti-inflammatory actions and are up-regulated in lymphocytes from ALS patients(Vincenzi et al., 2013). The A_3_R is a promising therapeutic target for inflammatory diseases (Jacobson et al., 2017) but these receptors have been mostly disregarded in what concerns ALS.

In conclusion, though one starts to envisage changes in the adenosinergic system at different stages of ALS progression, much more needs to be known before understanding the causal relationship of those changes and which of them can be targeted to develop novel therapeutics toward this devastating disease.

## Author Contributions

All authors listed have made a substantial, direct and intellectual contribution to the work, and approved it for publication.

## Conflict of Interest Statement

The authors declare that the research was conducted in the absence of any commercial or financial relationships that could be construed as a potential conflict of interest.
